# Complete chloroplast genome sequence of *Thermopsis turkestanica* Gand. (Leguminosae)

**DOI:** 10.1080/23802359.2020.1867017

**Published:** 2021-02-04

**Authors:** Pei-Pei Jiao, Xi Jiang, Wen-Rui Qu, Shan-He Zhang, Tian-Ge Yang, Zhi-Hua Wu

**Affiliations:** a Key Laboratory of Protection and Utilization of Biological Resources in Tarim Basin Xinjiang Production and Construction Corps, Tarim University, Alar, China; b College of Life Science, Tarim University, Alar, China; c College of Life Science and Technology of Huazhong Agricultural University, Wuhan, China; d College of Plant Science, Tarim University, Alar, China; e Hubei Provincial Key Laboratory for Protection and Application of Special Plant Germplasm in Wuling Area of China, College of Life Sciences, South-Central University for Nationalities, Wuhan, China

**Keywords:** *Thermopsis turkestanica*, chloroplast genome, evolution

## Abstract

*Thermopsis turkestanica* Gand. is a perennial herbaceous plant belonging to the genus *Thermopsis*, Leguminosae, and is mainly distributed in dry areas. Most of the species in this genus have high medicinal value. The complete chloroplast genome was reported in this study. The chloroplast genome with a total size of 149,551 bp consists of two inverted repeats (IRs, 24,159 bp) separated by a large single-copy region (LSC, 83,692 bp) and a small single-copy region (SSC, 17,541 bp). Further annotation revealed the chloroplast genome contains 110 genes, including 77 protein coding genes, 29 tRNA genes, and four rRNA genes. This information will be useful for study on the evolution and genetic diversity of *Thermopsis turkestanica* in the future.

*Thermopsis turkestanica* Gand. is a perennial herbaceous plant belonging to the genus *Thermopsis*, Leguminosae, and mainly distributed in the river valley and hillside of Xinjiang Tianshan Belt of China, it is also distributed in Mongolia, Altai, Kazakhstan, Uzbekistan, Turkmenistan, Kyrgyzstan, Tajikistan, and western Siberia of Russia. Most plants in this genus are frequently used as folk medicine in Northwest and Northeast China, such as the anti-cancer effect (Gao and Zhu 1999; Yildiz et al. 2020). In this study, to obtain the new insight into the phylogeny of *T. turkestanica*, we assembled and annotated the accurate chloroplast genome from sequenced data using Illumina HiSeq platform.

The materials of *T. turkestanica* in this study were collected from Aheqi County, Kizilsu Kirghiz Autonomous Prefecture, Xinjiang province of China (77°39.017′E, 40°49.949′N, 2464 m above sea level). The voucher specimen (TD-01531, *Thermopsis turkestanica* Gand.) was stored in the herbarium of Tarim University. The complete genomic DNA was extracted using CTAB method and sequenced using the Illumine HiSeq platform. First, we remove low quality sequences and joints from the raw data (SRR12880891), then quality control. The whole chloroplast genome was assembled using GetOrganelle (Jin et al. 2020). Then, the chloroplast gene structures were annotated using CPGAVAS2 (Shi et al. 2019) and PGA (Qu et al. 2019). The complete chloroplast genome was 149,551 bp (MW122835) and composed of two inverted repeats (IRs) of 24,159 bp each, which divide a large single-copy (LSC) region of 83,692 bp and a small single-copy (SSC) region of 17,541 bp, the average GC content was 36.41%. The chloroplast genomes encoded 110 functional genes, including 77 protein-coding genes, 29 tRNA genes, and four rRNA genes.

In our study, to explore the phylogenetic relationship of *T. turkestanica* within Leguminosae, additional 27 species from Leguminosae were studied. With the *Polygala japonica* and *Polygala tenuifolia* as the outgroups, the phylogenetic trees were built from the whole protein-coding gene matrix by maximum-likelihood (ML) and Bayesian inference (BI) (Figure 1). The ML tree was generated using IQ-TREE (Nguyen et al. 2015) based on the best model of TVM + F+R4 and 1000 bootstrap replicates, and BI analysis was performed in MrBayes-3.2.7. This result showed that the analyzed *T. turkestanica* was closer to the species of *Piptanthus concolor*. This information will provide the basis for the study of *Thermopsis turkestanica* in the future.

**Figure 1.**
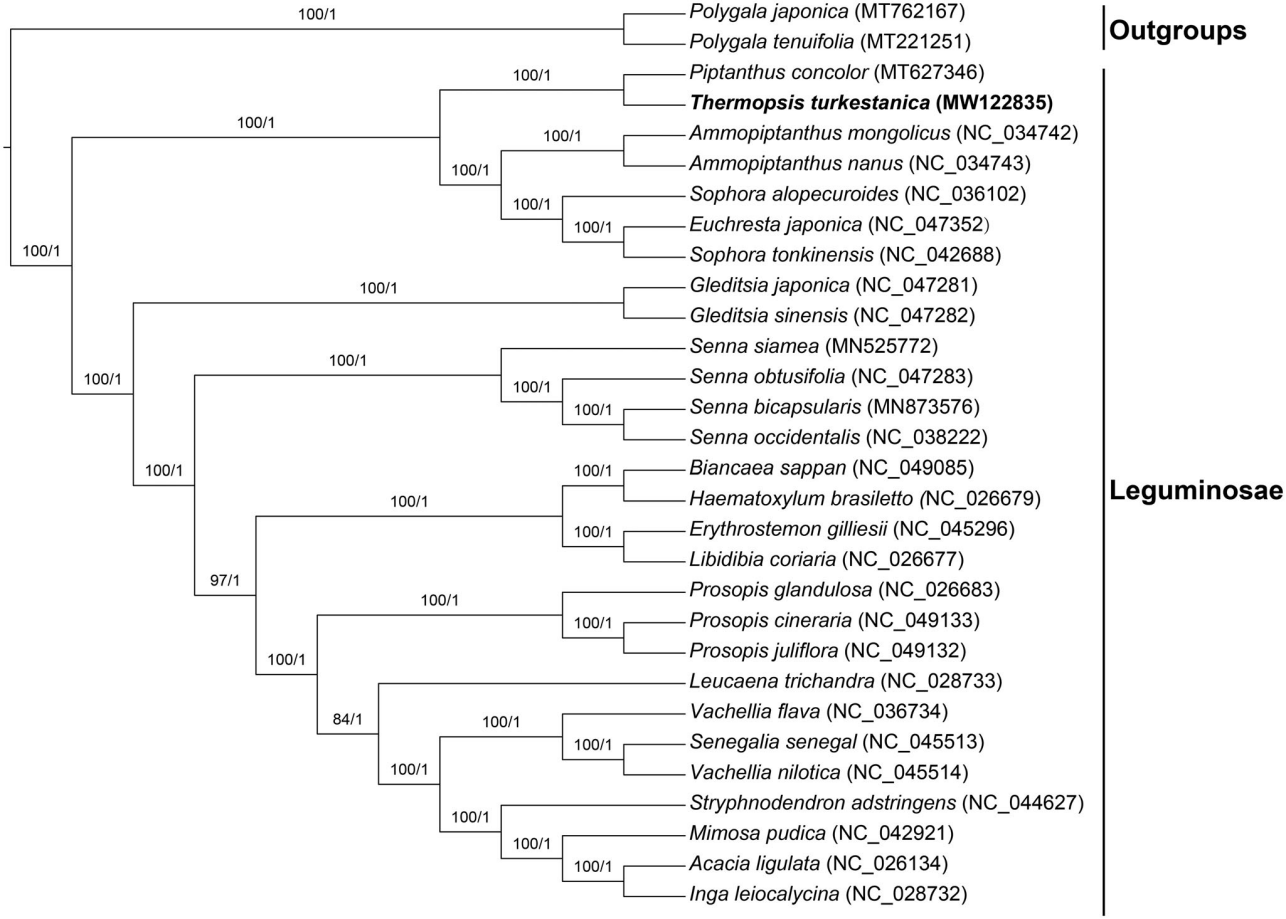
Phylogenetic tree reconstructed by maximum-likelihood (ML) and Bayesian inference (BI) analysis based on the whole chloroplast protein-coding genes of these 30 species.

## Data Availability

The genome sequence data that support the findings of this study are openly available in GenBank of NCBI at https://www.ncbi.nlm.nih.gov/ under the accession no. MW122835. The associated ‘BioProject’, ‘SRA’, and ‘Bio-Sample’ numbers are PRJNA670258, SRR12880891, and SAMN16491007, respectively.
